# Favorable outcome of neoadjuvant endocrine treatment than surgery‐first in female HR‐positive/HER2‐negative breast cancer patients—A NCDB analysis (2010–2016)

**DOI:** 10.1002/cam4.7244

**Published:** 2024-06-10

**Authors:** Peng Xu, Wen Luo, Jingjing Hu, Xiaobin Ma, Qian Hao, Wentao Hui, Zhangjian Zhou, Shuai Lin, Meng Wang, Hao Wu, Zhijun Dai, Huafeng Kang

**Affiliations:** ^1^ The Comprehensive Breast Care Center The Second Affiliated Hospital of Xi'an Jiaotong University Xi'an Shaanxi China; ^2^ Massachusetts General Cancer Center Boston Massachusetts USA; ^3^ Department of Biophysics, School of Basic Medical Sciences, Key Laboratory of Environment and Genes Related to Diseases Xi'an Jiaotong University Health Science Center Xi'an Shaanxi China; ^4^ Department of Breast Surgery The First Affiliated Hospital, College of Medicine, Zhejiang University Hangzhou China

**Keywords:** HR‐positive/HER2‐negative breast cancer, National Cancer Database, neoadjuvant endocrine treatment, overall survival, surgery

## Abstract

**Purpose:**

To assess the efficacy of neoadjuvant endocrine therapy in female HR‐positive/HER2‐negative breast cancer patients.

**Data and Methods:**

We identified female patients aged ≥18 years with cT1‐4N0‐XM0, HR(+), and HER2(−) breast cancer from the National Cancer Database. The patients who underwent surgery first were categorized as “surgery‐first,” while those who received NET before surgery were classified as “NET.” Propensity score‐matching, Cox proportional‐hazard model, variance inflation factors, and interaction analysis were employed to estimate the correlation between NET and survival outcomes.

**Results:**

Among 432,387 cases, 2914 NET patients and 2914 surgery‐first patients were matched. Compared with the surgery‐first group, the NET group received less adjuvant chemotherapy (*p* < 0.001). Furthermore, the NET group exhibited higher survival probabilities compared with the surgery‐first group (3 years: 91.4% vs. 82.1%; 5 years: 82.1% vs. 66.8%). Multivariate Cox analysis indicated that NET was associated with improved OS (surgery‐first vs. NET: HR 2.17, 95% CI: 1.93–2.44). Age over 55 years old, having public insurance, higher CDCC score, higher NSBR grade, ER(+)PR(−), and advanced clinical stage were related to worse OS (all *p* < 0.05). There was an interaction between age, race, income, and home and treatment regimen (all *p* < 0.05).

**Conclusion:**

NET may be a more effective treatment procedure than surgery‐first in female HR‐positive/HER2‐negative, non‐metastatic breast cancer patients. Future clinical studies with more detailed data will provide higher‐level evidence‐based data.

## 1 INTRODUCTION

Nowadays, neoadjuvant endocrine therapy (NET) has been used as an alternative to treat female HR(+) breast cancer patients who are not eligible for surgery or neoadjuvant chemotherapy (NCT) due to advanced age or frailty.^1^, ^2^, ^3^, ^4^

Over the years, numerous clinical trials recognized that NET was well‐tolerated and offered several possible advantages, such as reducing tumor size, downstaging, making unresectable tumors resectable,^5^, ^6^ and assessing tumor sensitivity to endocrine therapy.^7^, ^8^, ^9^, ^10^, ^11^ Moreover, NET can be administered orally, and thus, the treatment can be completed through outpatient visits without requiring hospitalization. Therefore, under the exhaustion of medical resources during the COVID‐19 epidemic, NET was recommended to overcome treatment (surgery or NCT) delays of several weeks to months in ER(+)HER2(−) breast cancer patients.^12^, ^13^, ^14^, ^15^, ^16^ Some articles reported that receiving NET treatment may offer a better prognosis than delaying surgery. However, other studies present a contrasting view, suggesting that NET may lead to delayed surgery, which can negatively affect prognosis.^17^, ^18^ The longer the surgery delay, the worse the overall outcome.^19^ However, Goldbach reported that a short duration of NET did not lead to a lower overall response rate based on the NCDB database.^13^ Furthermore, some trials and reviews investigated the efficacy of NET and NAC among HR‐positive/HER2‐negative breast cancer patients. Most of them reported similar pathologic response rates between the two neoadjuvant treatment strategies, and importantly, NET was related to lower adverse events and toxicity.

Today, the COVID epidemic has dissipated, and NET is no longer used as an alternative for delaying surgery or NCT during special periods. However, NET itself remains a promising treatment strategy that is worth further research. Therefore, we hope to use the large sample data provided by the NCDB to validate the advantages and disadvantages of NET compared to surgery plus adjuvant chemotherapy.

## 2 MATERIALS AND METHODS

### 2.1 Data source and population selection and data extraction

The data for this study were obtained from the National Cancer Database (NCDB) covering 2004 to 2016. The NCDB is a clinical oncology database that collects information from over 1500 Commission on Cancer‐accredited facilities in the United States. It provides extensive data on patient demographics, tumor characteristics, treatment patterns, and outcomes for both adult and pediatric cancer patients.^20^ It is invaluable, providing huge clinical samples and detailed clinical data for healthcare providers, researchers, policymakers, and other stakeholders to improve cancer diagnosis and prognosis.

The study included female patients aged 18 years and older diagnosed with clinical T1‐4N0‐XM0, clinical TNM stage 0‐III, HR‐positive/HER2‐negative breast cancer (histological code = 8000–8800) via histological and clinical confirmation. Patients with multiple primary tumors, those who did not undergo surgery, and those with critical missing information regarding treatment, follow‐up duration, or survival status were excluded.

For each case, the following individual characteristics were extracted: patient demographics (age, race, insurance status, income level, education level of high school, urbanization level of residence, and Charlson–Deyo (CDCC) score), tumor characteristics (laterality, Nottingham modification of the Scarff–Bloom–Richardson grading scheme (NSBR) grade, histologic grade, combined ER‐PR status, and clinical TNM stage), treatment procedures (surgery, chemotherapy, hormone therapy, radiation, response to neoadjuvant therapy, time between diagnosis and treatment initiation), length of follow‐up, and survival status.

The study categorized patients into two groups: “surgery‐first” and “NET.” “Surgery‐first” was defined as patients who: (1) underwent breast cancer surgery directly after diagnosis; (2) recorded as “systemic therapy after surgery” in item “RX_SUMM_SYSTEMIC_SUR_SEQ”; (3) recorded as “no neoadjuvant therapy” in item “CS Site‐Specific Factor 22”; (4) received chemotherapy and/or endocrine therapy along with other treatments post‐surgery. “NET” was defined as patients who: (1) initiated endocrine therapy before surgery; (2) received chemotherapy post‐surgery or not; (3) record as “systemic therapy before surgery” in item “RX_SUMM_SYSTEMIC_SUR_SEQ”; (4) record as “complete response (CR),” “partial response (PR),” “response noted but no mention if it was complete” or “partial and no response” in item “CS Site‐Specific Factor 22”; (5) Time elapsed between DX_HORMONE_STARTED_DAYS and DX_SURG_STARTED_DAYS was recognized as endocrine treatment duration.

### 2.2 Statistical analysis

Descriptive statistics were utilized to calculate frequencies and proportions. The chi‐square test was employed to compare the characteristics across various treatment groups. Propensity score‐matching (PSM) with a 1:1 ratio was conducted to minimize selection bias. Matching criteria were selected based on relevant clinical factors and previous research findings that might influence treatment decisions and patient survival. The covariates matched included age, race, insurance status, income level, education level of high school, urbanization level of residence, CDCC score, laterality, NSBR grade, histologic grade, combined ER‐PR status, cT, cN, clinical TNM stage. The primary endpoint of the study was overall survival (OS), estimated using Kaplan–Meier curves and log‐rank tests.

The variance inflation factor (VIF) was used to evaluate the multicollinearity among the variables in the regression model. Generally, VIFs exceeding four warrant further investigation, while VIFs exceeding 10 are signs of serious multicollinearity requiring correction. In this case, we choose to remove some of the violating predictors from the model to deal with multicollinearity. Univariable and multivariable Cox proportional hazard models were utilized to determine hazard ratios (HR) and corresponding 95% confidence intervals (CIs). Next, we conducted a multi‐factor adjusted interaction analysis to assess whether the effect of NET and surgery‐first were different between subgroups of selected covariates.

Statistical analysis was performed using R software (Version 4.3.2) and relevant R packages, including “CBCgrps,” “survival,” “survminer,” “MatchIt,” and “autoReg.” All p‐values were two‐sided, and a significance level of *p* < 0.05 was considered statistically significant.

## 3 RESULTS

### 3.1 Baseline characteristics of patients before and after PSM

**FIGURE 1:**
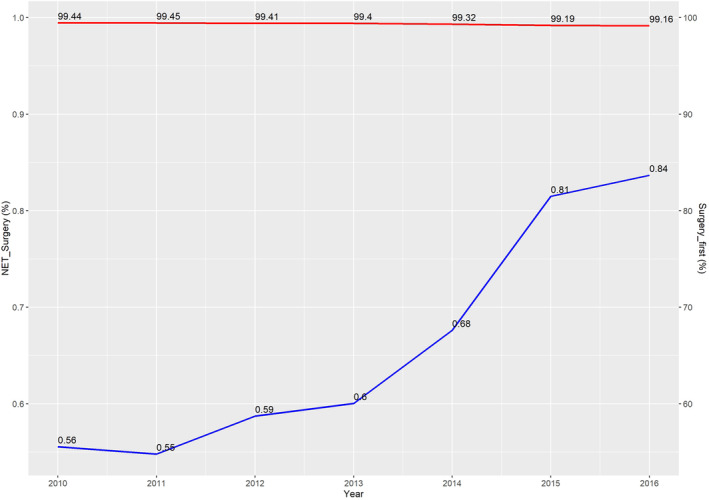
Trends of neoadjuvant endocrine treatment and surgery‐first by year.

A total of 432,387 cases diagnosed between 2010 and 2016 were eligible for this study. Among them, the NET group comprised 2914 (0.67%) patients, while surgery‐first group included 429,473(99.33%) patients. As shown in Figure 1, the utilization of NET increased steadily year by year, from 0.56% in 2010 to 0.84% in 2016, with the lowest rate (0.55%) in 2011. The median duration of endocrine therapy before surgery was 153 days, as illustrated in the histogram (Figure S1). Compared with patients who underwent surgery first, those who received NET were more likely to be older (*p* < 0.001), to have a higher score of CDCC (*p* < 0.001), and to have early clinical T/N/TNM stage (all *p* < 0.001) (Table S1). In addition, the surgery‐first group had a higher proportion of chemotherapy and radiotherapy after surgery compared with the NET group (all *p* < 0.001).

After PSM, 5828 patients were matched (NET group: 2919, surgery‐first group: 2919). The *p*‐values for all covariates included were greater than 0.05, indicating that propensity scores for the two groups significantly overlapped (Table 1). Compared with the surgery‐first group, the NET group was observed to have a higher likelihood of undergoing BCS (*p* < 0.001) and radiotherapy (*p* = 0.01) after surgery, while the surgery‐first group was more likely to receive adjuvant chemotherapy (27% vs. 4%, *p* < 0.001). Importantly, about 78% of patients reached partial or complete response after NET.

**TABLE 1:** Demographic and Clinical characteristics of HR‐positive/HER2‐negative breast cancer patients after PSM.

Variables		Total (*n* = 5828)	NET‐surgery (*n* = 2914)	Surgery‐first (*n* = 2914)	*p*
Age, *n* (%)	18–55	730 (13)	357 (12)	373 (13)	0.553
	55+	5098 (87)	2557 (88)	2541 (87)	
Race, *n* (%)	White	5010 (86)	2495 (86)	2515 (86)	0.89
	Black	556 (10)	285 (10)	271 (9)	
	Asian/PacificIslander	184 (3)	95 (3)	89 (3)	
	Other/Unknown	78 (1)	39 (1)	39 (1)	
Insurance, *n* (%)	No	100 (2)	51 (2)	49 (2)	0.881
	Public	3638 (62)	1820 (62)	1818 (62)	
	Private	1974 (34)	981 (34)	993 (34)	
	Unknown	116 (2)	62 (2)	54 (2)	
Income, *n* (%)	Less_than_$40,227	799 (14)	391 (13)	408 (14)	0.765
	$40,227‐50,353	940 (16)	478 (16)	462 (16)	
	$50,354‐63,332	1106 (19)	565 (19)	541 (19)	
	$63,333_or_more	2130 (37)	1048 (36)	1082 (37)	
	Unknown	853 (15)	432 (15)	421 (14)	
Highschool, *n* (%)	Highschool_Level_1	1531 (26)	751 (26)	780 (27)	0.908
	Highschool_Level_2	1736 (30)	868 (30)	868 (30)	
	Highschool_Level_3	1065 (18)	543 (19)	522 (18)	
	Highschool_Level_4	704 (12)	353 (12)	351 (12)	
	Unknown	792 (14)	399 (14)	393 (13)	
Home, *n* (%)	Metro	5015 (86)	2497 (86)	2518 (86)	0.716
	Rural	66 (1)	37 (1)	29 (1)	
	Urban	571 (10)	292 (10)	279 (10)	
	Unknown	176 (3)	88 (3)	88 (3)	
CDCC, *n* (%)	CDCC_Score_0	4592 (79)	2289 (79)	2303 (79)	0.754
	CDCC_Score_1	878 (15)	448 (15)	430 (15)	
	CDCC_Score_2	239 (4)	122 (4)	117 (4)	
	CDCC_Score_3	119 (2)	55 (2)	64 (2)	
Laterality, *n* (%)	Right	2952 (51)	1478 (51)	1474 (51)	0.937
	Left	2876 (49)	1436 (49)	1440 (49)	
Histologic grade, *n* (%)	Well	1644 (28)	834 (29)	810 (28)	0.782
	Moderately	3200 (55)	1581 (54)	1619 (56)	
	Poorly	647 (11)	326 (11)	321 (11)	
	Unknown	337 (6)	173 (6)	164 (6)	
Combined ER‐PR status, *n* (%)	ER(+)PR(+)	5303 (91)	2635 (90)	2668 (92)	0.343
	ER(+)PR(−)	516 (9)	274 (9)	242 (8)	
	ER(−)PR(+)	9 (0)	5 (0)	4 (0)	
NSBR grade, *n* (%)	Low grade	1847 (32)	933 (32)	914 (31)	0.66
	Medium grade	2904 (50)	1435 (49)	1469 (50)	
	High grade	549 (9)	271 (9)	278 (10)	
	Unknown	528 (9)	275 (9)	253 (9)	
cT, *n* (%)	cT1	1344 (23)	673 (23)	671 (23)	0.99
	cT2	3016 (52)	1512 (52)	1504 (52)	
	cT3	879 (15)	435 (15)	444 (15)	
	cT4	589 (10)	294 (10)	295 (10)	
cN, *n* (%)	cN0	4588 (79)	2295 (79)	2293 (79)	0.976
	cN1	1029 (18)	510 (18)	519 (18)	
	cN2	141 (2)	72 (2)	69 (2)	
	cN3	41 (1)	21 (1)	20 (1)	
	cNx	29 (0)	16 (1)	13 (0)	
Clinical TNM stage, *n* (%)	cStage1	1238 (21)	620 (21)	618 (21)	0.951
	cStage2	3678 (63)	1834 (63)	1844 (63)	
	cStage3	912 (16)	460 (16)	452 (16)	
Surgery, *n* (%)	Breast conservation surgery	3240 (56)	1750 (60)	1490 (51)	< 0.001
	Radical mastectomy	892 (15)	364 (12)	528 (18)	
	Simple mastectomy	1696 (29)	800 (27)	896 (31)	
Chemotherapy, *n* (%)	Chemotherapy	913 (16)	121 (4)	792 (27)	< 0.001
	None	4915 (84)	2793 (96)	2122 (73)	
Hormone Therapy, *n* (%)	Hormone therapy	5239 (90)	2914 (100)	2325 (80)	< 0.001
	None	589 (10)	0 (0)	589 (20)	
Radiotherapy, *n* (%)	Radiotherapy	3330 (57)	1722 (59)	1608 (55)	0.01
	None	2449 (42)	1169 (40)	1280 (44)	
	Unknown	49 (1)	23 (1)	26 (1)	
Response to NET, *n* (%)	Complete response (CR)	111 (2)	111 (4)	0 (0)	< 0.001
	No neoadjuvant	2914 (50)	0 (0)	2914 (100)	
	No response	641 (11)	641 (22)	0 (0)	
	Partial response (PR)	1346 (23)	1346 (46)	0 (0)	
	Response CR/PR	816 (14)	816 (28)	0 (0)	
Survival status, *n* (%)	Alive	4553 (78)	2466 (85)	2087 (72)	< 0.001
	Dead	1275 (22)	448 (15)	827 (28)	

Abbreviations: CDCC, Charlson‐Deyo score; cN, clinical N stage; cT, clinical T stage; NET, neoadjuvant endocrine therapy; NSBR‐, Nottingham modification of the Scarff‐Bloom‐Richardson grading scheme grade; PSM, Propensity score‐matching.

### 3.2 Survival analysis of matched cohort

**FIGURE 2:**
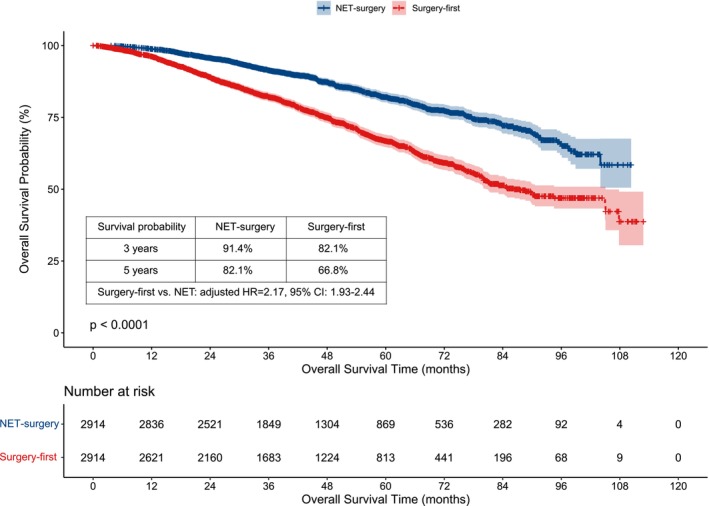
Kaplan–Meier survival of overall survival between NET and surgery‐first groups after PSM.

The median survival time was 43.17 (0.00–112.82) months in total patients, 41.51 (0.00–112.82) months in the NET group, and 44.41 (1.05–110.32) months in the surgery‐first group, respectively. The Kaplan–Meier curve showed that the three‐year and five‐year estimated OS probability of the NET group was higher than that of the surgery‐first group (3 years: 91.4% vs. 82.1%; 5 years: 82.1% vs. 66.8%) (Figure 2).

### 3.3 Multivariate analysis of matched cohort

Initially, the VIF was estimated, revealing significant multicollinearity among certain variables: cT and clinical stage, income and high school level, histologic grade, and NSBR grade. Therefore, variables including cT, cN, high school level, and histologic grade were excluded from the Cox regression analysis. Following this refinement, the VIF score was reevaluated, and the VIF values of all remaining variables were less than 2.

**FIGURE 3:**
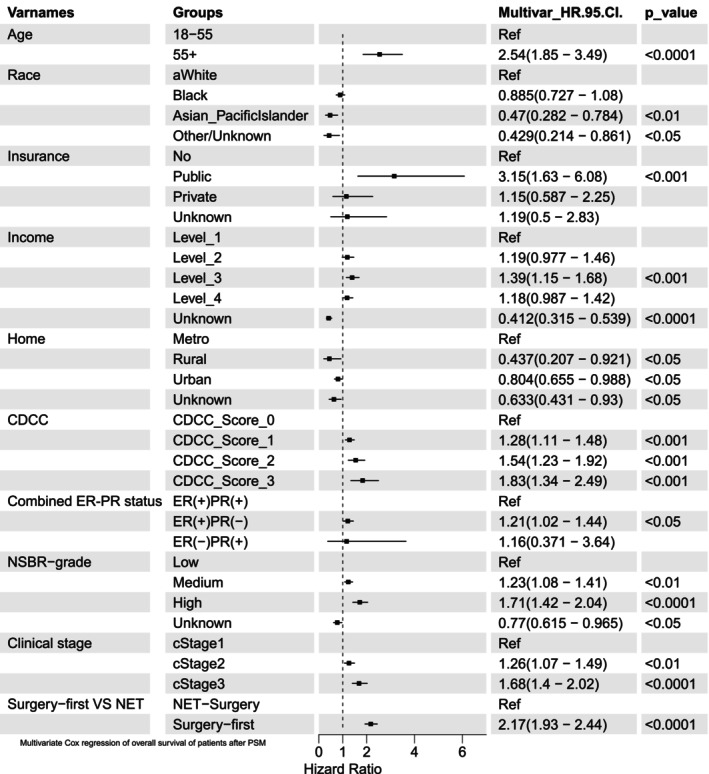
Forest plot of multivariate Cox regression of overall survival of patients after PSM.

The multivariate Cox analysis results were visualized in the forest plot (Figure 3) and detailed in Table 2. First, patients who received NET had a better prognosis than those who underwent surgery first (surgery‐first vs. NET: HR 2.17, 95% CI: 1.93–2.44). Asian and Pacific Islanders exhibited better OS (HR, 0.47; 95% CI, 0.282–0.784) compared to white individuals, and patients residing in rural or urban areas showed a more favorable prognosis than those in metropolitan areas (all *p* < 0.05). Second, several risk factors were significantly associated with worse OS, including age over 55 years (HR, 2.54, 95% CI: 1.85–3.49), having public medical insurance (Vs. no insurance HR 3.15, 95% CI: 1.63–6.08), having higher CDCC score (Vs. score 0, all *p* < 0.001), having higher NSBR grade (Vs. low grade, all *p* < 0.001), having ER(+)PR(−) (Vs. ER(+)PR(+), HR 1.21, 95% CI: 1.02–1.44, *p* < 0.05), and advanced clinical stage (all *p* < 0.001).

**TABLE 2:** Univariate and multivariate Cox regression analyses for overall survival of patients after PSM.

Variables	Groups	*n* (%)	Univar HR (95%CI)	*p*. value	Multivar HR (95%CI)	*p*. value
Age	18–55	730 (12.5%)	Ref		Ref	
	55+	5098 (87.5%)	4.54 (3.34–6.17)	0.0000	2.54 (1.85–3.49)	0.0000
Race	White	5010 (86.0%)	Ref		Ref	
	Black	556 (9.5%)	0.922 (0.764–1.11)	0.3970	0.885 (0.727–1.08)	0.2270
	Asian/PacificIslander	184 (3.2%)	0.352 (0.212–0.586)	0.0001	0.47 (0.282–0.784)	0.0038
	Other/Unknown	78 (1.3%)	0.473 (0.236–0.949)	0.0350	0.429 (0.214–0.861)	0.0173
Insurance	No	100 (1.7%)	Ref		Ref	
	Public	3638 (62.4%)	3.69 (1.92–7.12)	0.0001	3.15 (1.63–6.08)	0.0006
	Private	1974 (33.9%)	1.03 (0.529–2.02)	0.9220	1.15 (0.587–2.25)	0.6848
	Unknown	116 (2.0%)	1.14 (0.479–2.7)	0.7730	1.19 (0.5–2.83)	0.6956
Income	Less_than_$40,227	799 (13.7%)	Ref		Ref	
	$40,227‐50,353	940 (16.1%)	1.15 (0.947–1.4)	0.1580	1.19 (0.977–1.46)	0.0831
	$50,354‐63,332	1106 (19.0%)	1.26 (1.05–1.52)	0.0140	1.39 (1.15–1.68)	0.0008
	$63,333_or_more	2130 (36.5%)	1.07 (0.904–1.27)	0.4200	1.18 (0.987–1.42)	0.0684
	Unknown	853 (14.6%)	0.361 (0.277–0.471)	0.0000	0.412 (0.315–0.539)	0.0000
Home	Metro	5015 (86.1%)	Ref		Ref	
	Rural	66 (1.1%)	0.437 (0.208–0.919)	0.0290	0.437 (0.207–0.921)	0.0295
	Urban	571 (9.8%)	0.797 (0.653–0.973)	0.0256	0.804 (0.655–0.988)	0.0378
	Unknown	176 (3.0%)	0.565 (0.385–0.827)	0.0034	0.633 (0.431–0.93)	0.0199
CDCC	CDCC_Score_0	4592 (78.8%)	Ref		Ref	
	CDCC_Score_1	878 (15.1%)	1.29 (1.12–1.49)	0.0005	1.28 (1.11–1.48)	0.0009
	CDCC_Score_2	239 (4.1%)	1.98 (1.59–2.46)	0.0000	1.54 (1.23–1.92)	0.0002
	CDCC_Score_3	119 (2.0%)	2.43 (1.79–3.3)	0.0000	1.83 (1.34–2.49)	0.0001
Laterality	Right	2952 (50.7%)	Ref			
	Left	2876 (49.3%)	1.06 (0.948–1.18)	0.3180		
Combined ER‐PR status	ER(+)PR(+)	5303 (91.0%)	Ref			
	ER(+)PR(−)	516 (8.9%)	1.34 (1.13–1.59)	0.0008	1.21 (1.02–1.44)	0.0281
	ER(−)PR(+)	9 (0.2%)	1.38 (0.445–4.29)	0.5760	1.16 (0.371–3.64)	0.7963
NSBR grade	Low	1847 (31.7%)	Ref		Ref	
	Medium	2904 (49.8%)	1.28 (1.13–1.46)	0.0002	1.23 (1.08–1.41)	0.0021
	High	549 (9.4%)	2.13 (1.78–2.54)	0.0000	1.71 (1.42–2.04)	0.0000
	Unknown	528 (9.1%)	0.866 (0.693–1.08)	0.2070	0.77 (0.615–0.965)	0.0233
Clinical stage	cStage1	1238 (21.2%)	Ref		Ref	
	cStage2	3678 (63.1%)	1.36 (1.15–1.59)	0.0002	1.26 (1.07–1.49)	0.0052
	cStage3	912 (15.6%)	2.14 (1.79–2.57)	0.0000	1.68 (1.4–2.02)	0.0000
Surgery−first VS NET	NET‐surgery	2914 (50.0%)	Ref		Ref	
	Surgery‐first	2914 (50.0%)	2.08 (1.85–2.33)	0.0000	2.17 (1.93–2.44)	0.0000

Abbreviations: CDCC, Charlson–Deyo score; CI, confidence intervals; HR, hazard ratios; NET, neoadjuvant endocrine therapy; NSBR grade, Nottingham modification of the Scarff–Bloom–Richardson grading scheme grade; PSM, Propensity score‐matching.

Interaction analysis revealed significant interactions between the following factors and treatment (NET vs. surgery‐first): age (Surgery‐first*age55+, interaction *p* < 0.001), race (Surgery‐first*black, interaction *p* < 0.05), income (Surgery‐first*income level 3/4, interaction *p* < 0.001) and home (Surgery‐first*urban, interaction *p* < 0.05). No interaction was observed between the other variables, like insurance, CDCC score, combined ER‐PR status, NSBR grade, and clinical stage (all interactions *p* > 0.05).

## 4 DISCUSSION

In this research, we observed a high response rate of 78% to NET, and the NET group had higher BCS rates and 3/5 years survival rates than surgery‐first. What is more, NET treatment exhibited a favorable survival outcome compared to surgery‐first. These findings are consistent with other clinical trials. As early as 2005, a multicenter, double‐blind, randomized trial (IMPACT) reported that neoadjuvant anastrozole and tamoxifen is effective and well‐tolerated in ER‐positive operable breast cancer in postmenopausal women.^21^ In the following years, some trials compared the effectiveness of NET and NCT in HR‐positive/HER2–negative breast cancer. They observed similar clinical and radiological responses and BCS rates, and NET exhibited lower side effects.^7^, ^22^, ^23^ Recently, some RCT also suggested that NET may be used as standard treatment for postmenopausal patients with ER+ BC, but whether adjuvant chemotherapy is necessary remains unknown and may depend on the response rate to NET.^24^, ^25^, ^26^ Furthermore, another RCT study reported that NET may not be equivalent to NCT in premenopausal patients.^27^ In addition, some cohort studies also found that NET was well‐tolerated and achieved similar or better effectiveness.^9^, ^28^, ^29^, ^30^ Moreover, many studies based on NCDB databases hold the same views and conclusions, such as Kantor,^31^ Stafford,^32^ Chiba,^33^ and Tamirisa.^34^ While these studies unanimously support the role of NET in reducing pathological stage, axillary lymph node staging, and increasing BCS rates, they do not analyze the impact of treatment on survival prognosis.

It is obvious that NET treatment will delay the time of surgical treatment, which may increase the rate of BCS surgery, or it may lead to disease progression due to delayed surgery; how to balance the pros and cons. In 2016, Bleicher et al. conducted a cohort study utilizing the SEER database and discovered that a prolonged time to surgery is linked to a poorer prognosis, whereas a shortened delay did not show significant negative effects.^19^ Similarly, a new study in 2021 utilizing the NCDB database and discovered progressively worse OS with increasing surgery delay among patients who underwent upfront surgery.^16^ There are also studies that suggest that under special circumstances, like during a coronavirus epidemic, NET has been considered as an alternative to doing nothing, even though its efficacy remains uncertain.^12^, ^13^ Moreover, although number of researches indicated that NET was well‐tolerated and achieved similar effectiveness to NCT in postmenopausal women, it remains challenging to determine the appropriate patients for NET. Several studies suggested that patients with low Ki67 levels respond better to NET.^23^, ^29^, ^30^, ^35^, ^36^ Prospect traffic reported that pathological response is a favorable prognostic factor following NET.^37^ Additionally, other tools such as SET2,3,^38^ Oncotype Dx Breast Recurrence Score,^39^ and Preoperative Endocrine Prognostic Index (PEPI)^26^ have been validated to predict response to endocrine therapy.

In contrast to other studies that also utilized the NCDB database, our research primarily highlights the association of neoadjuvant endocrine therapy with favorable outcomes. This could be attributed to the fact that the populations we included were well‐matched and had consistent baseline characteristics. For instance, in the Goldbach study,^40^ the samples displayed statistically significant differences, prompting the author to contemplate these distinctions as a potential factor contributing to variations in survival rates. Additionally, there were contradictions and ambiguities in the entries provided by the NCDB database, which posed challenges in accurately screening the target sample. Therefore, our study implemented stricter inclusion criteria, particularly in defining NET and the “surgery‐first” approach. Therefore, the present study developed stricter inclusion criteria, especially the definition of NET and surgery‐first. Instead of relying solely on the order of endocrine therapy and surgery, criteria such as the “system‐surgical treatment sequence”, “surgical start time”, “endocrine therapy start time”, “chemotherapy start time”, “whether to receive surgical treatment” “whether to receive endocrine therapy,” “whether to receive chemotherapy,” etc., were considered. As a result, although the number of included samples was reduced in this study, the data obtained were more authentic and reliable.

The limitations of this study mainly stem from the data obtained from the NCDB database. Some important variables, such as the dose and type of endocrine drugs, ER/PR positivity degree, Ki67 expression status, menstrual status, number of births, and family history of malignancy, were not available in database. Additionally, inconsistent data on treatment‐related variables made it challenging to accurately screen the target population, possibly leading to departure from real‐world scenarios.

## 5 CONCLUSION

NET may potentially be more effective than surgery‐first in female HR‐positive/HER2‐negative breast cancer patients without distant metastasis. However, it is essential to note that NET may not be suitable for all HR(+)/HER2(−) breast cancer patients, and treatment decisions should be individualized based on specific patient circumstances and pathological characteristics. Future clinical studies with more detailed disease data will provide more precise data to identify the populations that will benefit the most. For patients who have undergone NET, postoperative adjuvant therapy and rigorous follow‐up in accordance with guideline standards are crucial.

## AUTHOR CONTRIBUTIONS

**Peng Xu:** Conceptualization (equal); data curation (lead); formal analysis (equal); writing – original draft (equal); writing – review and editing (lead). **Wen Luo:** Writing – original draft (equal); writing – review and editing (equal). **Jingjing Hu:** Data curation (equal); methodology (equal); resources (equal); software (equal). **Xiaobin Ma:** Conceptualization (equal); methodology (equal); supervision (equal); writing – review and editing (equal). **Qian Hao:** Investigation (equal); writing – review and editing (equal). **Wentao Hui:** Investigation (equal); methodology (equal). **Zhangjian Zhou:** Investigation (equal); methodology (equal). **Shuai Lin:** Investigation (equal); methodology (equal). **Meng Wang:** Investigation (equal); validation (equal). **Hao Wu:** Funding acquisition (equal); project administration (equal); supervision (equal). **Zhijun Dai:** Resources (equal); supervision (equal). **Huafeng Kang:** Conceptualization (equal); funding acquisition (equal); project administration (equal); supervision (equal).

## FUNDING INFORMATION

This study was supported in part by the key research and development program of Shaanxi Province (2022KW‐01 and 2022SF‐001) and “Basic‐clinical Cross‐disciplinary Plan” of Xi'an Jiaotong University, China (No. YXJLRH2022056).

## CONFLICT OF INTEREST STATEMENT

There is no conflict of interest exists in this manuscript.

## ETHICS STATEMENT

Patient consent was not required as this project utilized a deidentified database.

## Supporting information


Figure S1.

Table S1.

## Data Availability

The authors confirm that the data supporting the findings of this study are available within the article and its supplementary materials. Further inquiries can be directed to the corresponding authors.
