# Supplementary figures and images for: Favorable outcome of neoadjuvant endocrine treatment than surgery‐first in female HR‐positive/HER2‐negative breast cancer patients—A NCDB analysis (2010–2016)
Source: Cancer Med. 2024 Jun 10;13(11):e7244. doi: 10.1002/cam4.7244 (PMC11165171; doi:10.1002/cam4.7244)

Histogram of NET duration(days)

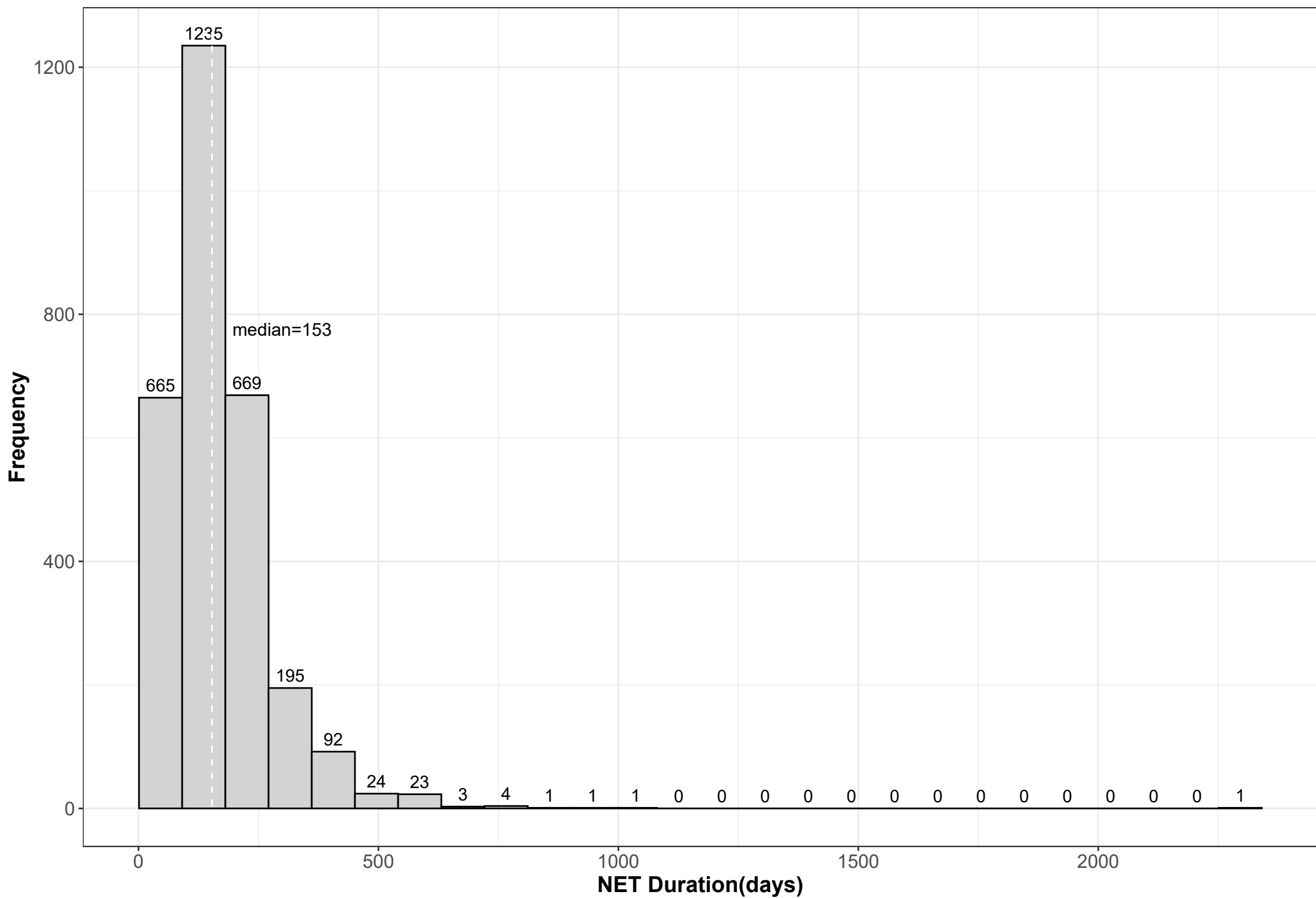

Supplement: Supplementary file 1 — Figure S1. [file CAM4-13-e7244-s001.pdf]
