# Supplementary material for: Favorable outcome of neoadjuvant endocrine treatment than surgery‐first in female HR‐positive/HER2‐negative breast cancer patients—A NCDB analysis (2010–2016)
Source: Cancer Med. 2024 Jun 10;13(11):e7244. doi: 10.1002/cam4.7244 (PMC11165171; doi:10.1002/cam4.7244)
Supplement: Supplementary file 2 — Table S1. [file CAM4-13-e7244-s002.docx]

# TABLE S1. Demographic and Clinical characteristics of total patients

| **Variables** | | **Total (n = 432387)** | **NET-Surgery (n = 2914)** | **Surgery-first (n = 429473)** | **p** |
| --- | --- | --- | --- | --- | --- |
| Age, n (%) | 18-55 | 126007 (29) | 357 (12) | 125650 (29) | < 0.001 |
|  | 55+ | 306380 (71) | 2557 (88) | 303823 (71) |  |
| Race, n (%) | White | 371816 (86) | 2495 (86) | 369321 (86) | 0.096 |
|  | Black | 37757 (9) | 285 (10) | 37472 (9) |  |
|  | Asian PacificIslander | 15948 (4) | 95 (3) | 15853 (4) |  |
|  | Unknown | 6866 (2) | 39 (1) | 6827 (2) |  |
| Insurance, n (%) | No | 6071 (1) | 51 (2) | 6020 (1) | < 0.001 |
|  | Public | 194917 (45) | 1820 (62) | 193097 (45) |  |
|  | Private | 222900 (52) | 981 (34) | 221919 (52) |  |
|  | Unknown | 8499 (2) | 62 (2) | 8437 (2) |  |
| Income, n (%) | Less_than_$40,227 | 54704 (13) | 391 (13) | 54313 (13) | 0.004 |
|  | $40,227-50,353 | 75594 (17) | 478 (16) | 75116 (17) |  |
|  | $50,354-63,332 | 88446 (20) | 565 (19) | 87881 (20) |  |
|  | $63,333_or_more | 158640 (37) | 1048 (36) | 157592 (37) |  |
|  | Unknown | 55003 (13) | 432 (15) | 54571 (13) |  |
| Highschool, n (%) | Highschool_Level_1 | 115988 (27) | 751 (26) | 115237 (27) | 0.007 |
|  | Highschool_Level_2 | 128856 (30) | 868 (30) | 127988 (30) |  |
|  | Highschool_Level_3 | 86828 (20) | 543 (19) | 86285 (20) |  |
|  | Highschool_Level_4 | 49606 (11) | 353 (12) | 49253 (11) |  |
|  | Unknown | 51109 (12) | 399 (14) | 50710 (12) |  |
| Home, n (%) | Metro | 365789 (85) | 2497 (86) | 363292 (85) | 0.002 |
|  | Rural | 6403 (1) | 37 (1) | 6366 (1) |  |
|  | Urban | 50400 (12) | 292 (10) | 50108 (12) |  |
|  | Unknown | 9795 (2) | 88 (3) | 9707 (2) |  |
| CDCC, n (%) | CDCC_Score_0 | 357845 (83) | 2289 (79) | 355556 (83) | < 0.001 |
|  | CDCC_Score_1 | 58851 (14) | 448 (15) | 58403 (14) |  |
|  | CDCC_Score_2 | 11675 (3) | 122 (4) | 11553 (3) |  |
|  | CDCC_Score_3 | 4016 (1) | 55 (2) | 3961 (1) |  |
| Laterality, n (%) | Right | 214863 (50) | 1478 (51) | 213385 (50) | 0.393 |
|  | Left | 217323 (50) | 1436 (49) | 215887 (50) |  |
|  | Unknown | 201 (0) | 0 (0) | 201 (0) |  |
| Histologic grade, n (%) | Well | 135189 (31) | 834 (29) | 134355 (31) | < 0.001 |
|  | Moderately | 210892 (49) | 1581 (54) | 209311 (49) |  |
|  | Poorly | 67669 (16) | 326 (11) | 67343 (16) |  |
|  | Undifferentiated | 304 (0) | 0 (0) | 304 (0) |  |
|  | Unknown | 18333 (4) | 173 (6) | 18160 (4) |  |
| Combined ER-PR status, n (%) | ER(+)PR(+) | 386856 (89) | 2635 (90) | 384221 (89) | < 0.001 |
|  | ER(+)PR(-) | 42272 (10) | 274 (9) | 41998 (10) |  |
|  | ER(-)PR(+) | 3259 (1) | 5 (0) | 3254 (1) |  |
| BR_grade, n (%) | Low BR-grade | 144084 (33) | 933 (32) | 143151 (33) | < 0.001 |
|  | Medium BR-grade | 202701 (47) | 1435 (49) | 201266 (47) |  |
|  | High BR-grade | 62903 (15) | 271 (9) | 62632 (15) |  |
|  | Unknown | 22699 (5) | 275 (9) | 22424 (5) |  |
| cT, n (%) | cT1 | 329469 (76) | 673 (23) | 328796 (77) | < 0.001 |
|  | cT2 | 91795 (21) | 1512 (52) | 90283 (21) |  |
|  | cT3 | 8972 (2) | 435 (15) | 8537 (2) |  |
|  | cT4 | 2151 (0) | 294 (10) | 1857 (0) |  |
| cN, n (%) | cN0 | 398185 (92) | 2295 (79) | 395890 (92) | < 0.001 |
|  | cN1 | 27467 (6) | 510 (18) | 26957 (6) |  |
|  | cN2 | 3259 (1) | 72 (2) | 3187 (1) |  |
|  | cN3 | 1154 (0) | 21 (1) | 1133 (0) |  |
|  | cNx | 2322 (1) | 16 (1) | 2306 (1) |  |
| Clinical TNM stage, n (%) | cStage0 | 399 (0) | 0 (0) | 399 (0) | < 0.001 |
|  | cStage1 | 317530 (73) | 620 (21) | 316910 (74) |  |
|  | cStage2 | 105667 (24) | 1834 (63) | 103833 (24) |  |
|  | cStage3 | 8791 (2) | 460 (16) | 8331 (2) |  |
| Surgery, n (%) | Breast conservation surgery | 296050 (68) | 1750 (60) | 294300 (69) | < 0.001 |
|  | Radical mastectomy | 33651 (8) | 364 (12) | 33287 (8) |  |
|  | Simple mastectomy | 102686 (24) | 800 (27) | 101886 (24) |  |
| Chemotherapy, n (%) | Chemotherapy | 105904 (24) | 121 (4) | 105783 (25) | < 0.001 |
|  | None | 326483 (76) | 2793 (96) | 323690 (75) |  |
| Hormone Therapy, n (%) | Hormone Therapy | 372411 (86) | 2914 (100) | 369497 (86) | < 0.001 |
|  | None | 59976 (14) | 0 (0) | 59976 (14) |  |
| Radiotherapy, n (%) | Radiotherapy | 287976 (67) | 1722 (59) | 286254 (67) | < 0.001 |
|  | None | 142948 (33) | 1169 (40) | 141779 (33) |  |
|  | Unknown | 1463 (0) | 23 (1) | 1440 (0) |  |
| Response to NET, n (%) | Complete response (CR) | 111 (0) | 111 (4) | 0 (0) | < 0.001 |
|  | No neoadjuvant | 429473 (99) | 0 (0) | 429473 (100) |  |
|  | No response | 641 (0) | 641 (22) | 0 (0) |  |
|  | Partial response (PR) | 1346 (0) | 1346 (46) | 0 (0) |  |
|  | Response CR/PR | 816 (0) | 816 (28) | 0 (0) |  |
| Survival status, n (%) | Alive | 400621 (93) | 2466 (85) | 398155 (93) | < 0.001 |
|  | Dead | 31766 (7) | 448 (15) | 31318 (7) |  |

Abbreviations: NET: neoadjuvant endocrine therapy; CDCC: Charlson-Deyo score; NSBR grade: Nottingham modification of the Scarff-Bloom-Richardson grading scheme grade; cT: clinical T stage; cN: clinical N stage.
